# Morphological and Geochemical Evidence of Eumelanin Preservation in the Feathers of the Early Cretaceous Bird, *Gansus yumenensis*

**DOI:** 10.1371/journal.pone.0025494

**Published:** 2011-10-13

**Authors:** Holly E. Barden, Roy A. Wogelius, Daqing Li, Phillip L. Manning, Nicholas P. Edwards, Bart E. van Dongen

**Affiliations:** 1 School of Earth Atmospheric and Environmental Sciences and Williamson Research Centre for Molecular Environmental Science, University of Manchester, Manchester, United Kingdom; 2 Gansu Geological Museum, Lanzhou, People's Republic of China; 3 Department of Earth and Environmental Sciences, University of Pennsylvania, Philadelphia, Pennsylvania, United States of America; Raymond M. Alf Museum of Paleontology, United States of America

## Abstract

Recent studies have shown evidence for the preservation of colour in fossilized soft tissues by imaging melanosomes, melanin pigment containing organelles. This study combines geochemical analyses with morphological observations to investigate the preservation of melanosomes and melanin within feathers of the Early Cretaceous bird, *Gansus yumenensis*. Scanning electron microscopy reveals structures concordant with those previously identified as eumelanosomes within visually dark areas of the feathers but not in lighter areas or sedimentary matrices. Fourier transform infrared analyses show different spectra for the feathers and their matrices; melanic functional groups appear in the feather including carboxylic acid and ketone groups that are not seen in the matrix. When mapped, the carboxylic acid group absorption faithfully replicates the visually dark areas of the feathers. Electron Paramagnetic Resonance spectroscopy of one specimen demonstrates the presence of organic signals but proved too insensitive to resolve melanin. Pyrolysis gas chromatography mass spectrometry shows a similar distribution of aliphatic material within both feathers that are different from those of their respective matrices. In combination, these techniques strongly suggest that not only do the feathers contain endogenous organic material, but that both geochemical and morphological evidence supports the preservation of original eumelanic pigment residue.

## Introduction

**Figure 1:**
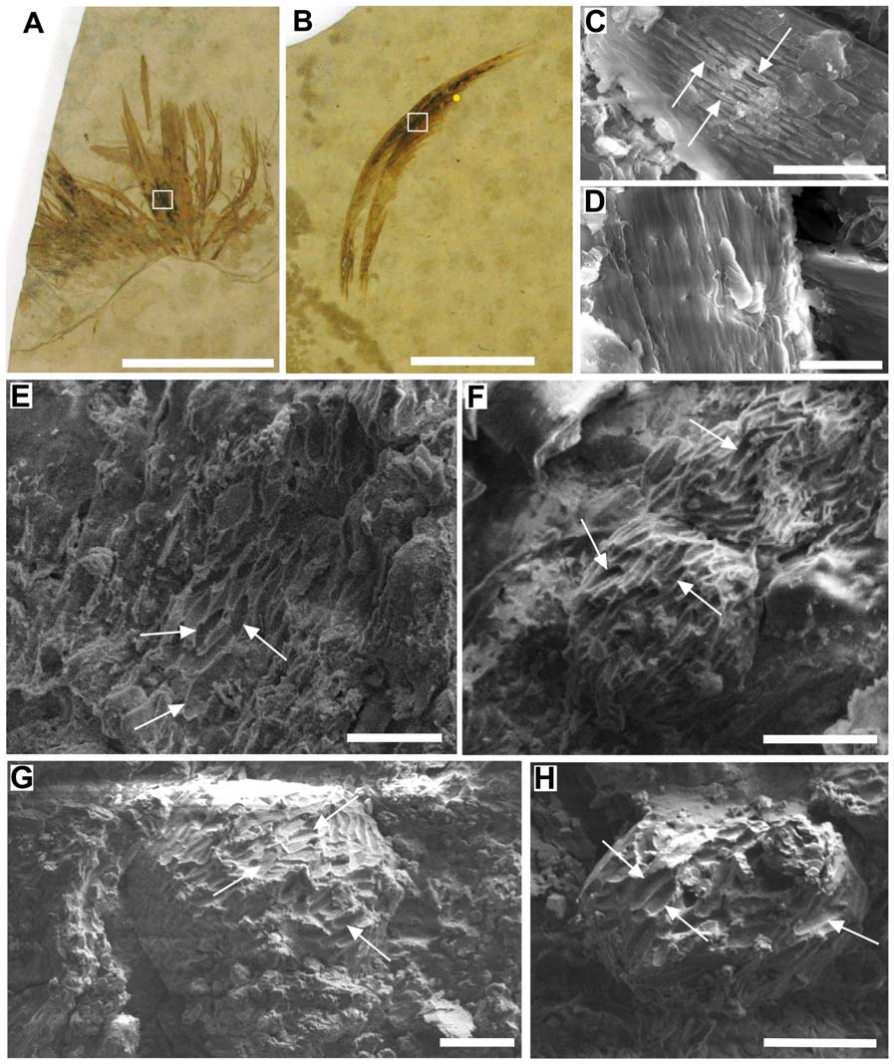
Visual and SEM images showing the presence of melanosomes in *Gansus yumenensis* and extant feathers. Isolated feathers (A) MGSF318 and (B) MGSF317, with SEM images of the fractured surfaces of (C) an extant Marabou stork feather and (D) a White-naped Crane feather, and the dark areas of MGSF317 (E-F) and MGSF318 (G-H). Eumelanosomes present within the extant Marabou stork feather (C) and the elongate mouldic structures interpreted as eumelanosomes in the fossil feathers (E-H) are highlighted with white arrows. Scale bars represent 2 cm (A-B), 5 µm (C–D) and 2 µm (E–H). The fossil feather SEM images were taken from the areas indicated by the white boxes, and the yellow dot in (B) represents the approximate area where the FTIR map discussed below is taken from.

Melanin is a common chemical pigment found ubiquitously throughout the natural world [1], [2]. As well as having a key role in display [3] and camouflage [1], melanin also provides UV protection [4], [5], thermoregulation [1], sequestration of potentially toxic metal ions [6], [7] and acts as a free radical sink [5], [6]. Modern studies of animal pigmentation provide insights to behavior, life history and evolution [3] that have typically been thought to be beyond the realm of palaeontology. Recent studies of fossil feathers [8]–[12] and a theropod dinosaur integument [8] however have shown evidence of microscopic structures that have been interpreted as melanosomes, the melanin containing organelles in extant organisms. This interpretation is based on cogent arguments regarding size, form [8]–[12], spatial arrangement [9], organisation [10] and apparent embedding within soft tissue [8] for the presumed melanosomes. Whilst these arguments are somewhat persuasive, the problem remains that bacteria are capable of replicating each one of these characteristics. This is a particular problem when trying to identify pheomelanosomes; their spheroid shape and lack of distinctive spatial organization make them even harder to distinguish from similarly sized coccoid bacteria. In addition, many modern bacteria are not only the same shape but also the same size as purported eumelanosome structures. Rod shaped bacillus bacteria range in length from 0.5 to 20 µm [13], and the average diameter of coccus bacteria is 0.5 [13] to 1.5 µm [14]. In a previous investigation on a fossil feather such structures were indeed thought to be bacteria covered with a glycocalyx (Figure 1a [15]), however in light of the recent studies on fossil feathers this assessment has been re-evaluated and the structures have since been identified as melanosomes surrounded by the remains of decaying β-keratin fibres [9]. In addition, some images of purported melanosomes clearly display the alveolar structure that is characteristic of microbial mats (Figure 1b,c [8]) [16], [17]. The fact that melanosomes are only found within darker areas of fossilized feathers is probably not coincidental, though it is possible that this pattern could be replicated by bacteria, as there are still conflicting reports about whether feather degrading bacteria preferentially feed on melanin or not [18]–[20].

This study combines both morphological (imaging) and organic geochemical techniques to analyse feathers from the early Cretaceous Chinese bird *Gansus yumenensis*. The results are compared to those of extant feathers and a standard melanin sample to try and determine the extent of organic preservation within the fossil feathers and whether any geochemical evidence of melanic or bacterial biomarkers can be identified.

## Materials and Methods

### Samples

The two fossil feathers are attributed to the Lower Cretaceous (early Aptian/Aptian, 125 to 112 Ma) amphibious bird *Gansus yumenensis* [21], [22]. Both specimens (MGSF318 and MGSF317, Fig. 1A and B respectively) were discovered in the Xiagou Formation near Changma in Gansu Province of northwestern China [21]. Originally from the Gansu Geological Museum, the fossils are currently housed in the collections of the School of Earth Atmospheric and Environmental Sciences, University of Manchester, and will be returned to China after research. Both specimens have had minimum handling, MGSF317 has not been touched with any glue or consolidants, there is a thin layer of consolidant (*cf* polyvinal butyral) on the underside of the matrix in MGSF318 though nothing compromising the chemical integrity of the fossil itself. Both specimens were handled with gloves at all times and stored in foil envelopes within sealed bags to minimize contamination. Samples of extant black (Marabou Stork (*Leptoptilos crumeniferus*)) and white (White-naped Crane (*Grus vipio*)) feathers were supplied from Birdland in Gloucestershire. Natural melanin from *Sepia officinalis* was supplied by Sigma-Aldrich. All fossil material, extant samples and standards were stored in isolation of one another, reducing possible cross-contamination.

### Sample preparation and analyses

All samples were analysed by variable pressure field emission gun scanning electron microsopy (VP-FEG-SEM) with energy dispersive X-ray spectrometry (EDS), Fourier transform infrared spectroscopy (FTIR), electron paramagnetic resonance (EPR) and pyrolysis gas chromatography mass spectrometry (Py-GCMS). VP-FEG-SEM provides microscopic analysis of the morphology of the samples and allows the visual identification of potential melanosomes without the need for coating the sample or subjecting it to high vacuum conditions as would be the case with traditional electron microscopes. EDS, FTIR, EPR and Py-GCMS are all geochemical techniques that give information about the elemental composition, functional groups, organic free radicals and involatile macromolecular complexes, respectively, of a sample. EPR and Py-GCMS are both destructive techniques requiring additional sample preparation of the extant feathers and fossil material. Extant feather samples were finely chopped into small pieces and were prepared for FTIR analysis by cleaning in distilled water in an ultrasonic bath for 10 minutes 3 times and then air-dried without heating. Powdered fossil material was obtained by carefully scraping fossil and matrix samples from the main block under negative pressure from a flow hood. Sterile scalpels were used in both cases and gloves were worn at all times.

### Scanning electron microscopy (SEM)/Energy dispersive X-ray spectrometry (EDS)

All specimens were analysed using variable pressure-field emission gun-scanning electron microscopy (VP-FEG-SEM). Extant specimens were prepared according to the technique in Zhang et al. [8], though they were not coated, and then imaged using an FEI XL30 instrument. Secondary electron images were taken at 10.0 keV, at a working distance of 10 mm. Elemental composition was analysed using an EDAX energy dispersive X-Ray spectrometer (EDS). Fossil specimens were analysed using a Zeiss Supra40VP instrument. Secondary electron images were taken at low accelerating voltage (2–3 keV) with a working distance of 9–10 mm. Standardless EDS spectroscopy was carried out using an Oxford Instruments machine to examine elemental composition via point analyses. All EDS spectra were taken at an accelerating voltage of 15 keV, a working distance of 15 mm, and were collected for 100 s. Errors on the standardless EDS analyses are estimated to be approximately 30% of the value reported.

### Fourier transform infrared spectroscopy (FTIR)

Spectra from the fossil specimens were taken using a Spotlight 400 Perkin Elmer FTIR Imaging System (wavenumber range 4000 to 800 cm^−1^). Point analyses and maps were collected in Attenuated Total Reflectance mode (ATR) with a 20×20 µm aperture and 4 cm^−1^ resolution; all final spectra were an average of 10 scans and were background subtracted. Maps are composed of point analyses taken in an 18×20 grid with 10 spectral scans per point. Extant samples were analysed using a Bio-Rad 6000-IR system (wavenumber range 4000 to 900 cm^−1^). Spectra were taken with a 2 cm^2^ aperture and 4 cm^−1^ resolution; final spectra were an average of 16 scans (20 for natural *S. officinalis* melanin). All spectra and maps were background subtracted. Organic peak assignments were made using the Bio-Rad KnowItAll Informatics System 8.2 Multi-Technique database. Inorganic peaks were assigned using reference mineral spectra from Russell et al. 1987 [23].

### Electron paramagnetic resonance (EPR)

X-band (9 GHz) Electron Spin Resonance spectroscopy was carried out at room temperature on an EMX Spectrometer using a High Q X-band HSW0541 cavity. All sample tubes were pre-furnaced at 400°C and stored in foil in order to avoid organic contamination. Due to the small samples sizes only MGSF318 was analysed with EPR spectra were collected for 125 scans. Power was varied to maintain line shape and to avoid saturation. Spectra were recorded with an attenuation of 24 dB (equivalent to 0.8 mW). All spectra were background subtracted. Magnetic field correction used the Bruker standard Strong Pitch (g = 2.0028) and the measured frequency was recorded by an internal frequency counter in the microwave bridge. g values are calculated by dividing the product of Planck's constant (*h*) and the frequency of the incident radiation (ν) by the product of the Bohr magneton (β_e_) with the magnetic induction of the magnetic field at resonance (B_r_): g = (*hν*)/(β_e_B_r_)

### Pyrolysis gas chromatography mass spectrometry (Py-GCMS)

Extant feather, fossil and melanin samples were analysed by normal flash pyrolysis GCMS. The samples were pyrolysed using a CDS (Chemical Data Systems) 5200 series pyroprobe pyrolysis unit by heating at 600°C for 20 s to fragment macromolecular components. These fragments were then analysed using an Agilent 7890A gas chromatograph fitted with a HP-5 fused column (J+W Scientific; 5% diphenyl-dimethylpolyolsiloxane; 30 m, 0.32 mm i.d., 0.25 µm film thickness) coupled to an Agilent 5975C MSD single quadrupole Mass Spectrometer operated in electron ionization (EI) mode (scanning a range of *m/z* 25-650 at 1 scan s^−1^ with a 4 minute solvent delay; ionization energy 70eV). The pyrolysis transfer line and injector port temperatures were set at 350°C, the heated interface at 280°C, the EI source at 230°C and the MS quadrupole at 150°C. Helium was used as the carrier gas and the samples were introduced in split mode in a ratio of 2∶1. The oven was programmed from 40°C (held for 4 minutes) to 320°C at 4°C min^−1^ and held at this temperature for 5 minutes. Compounds were identified by comparison with spectra from the literature.

## Results

### SEM/EDS

**Figure 2:**
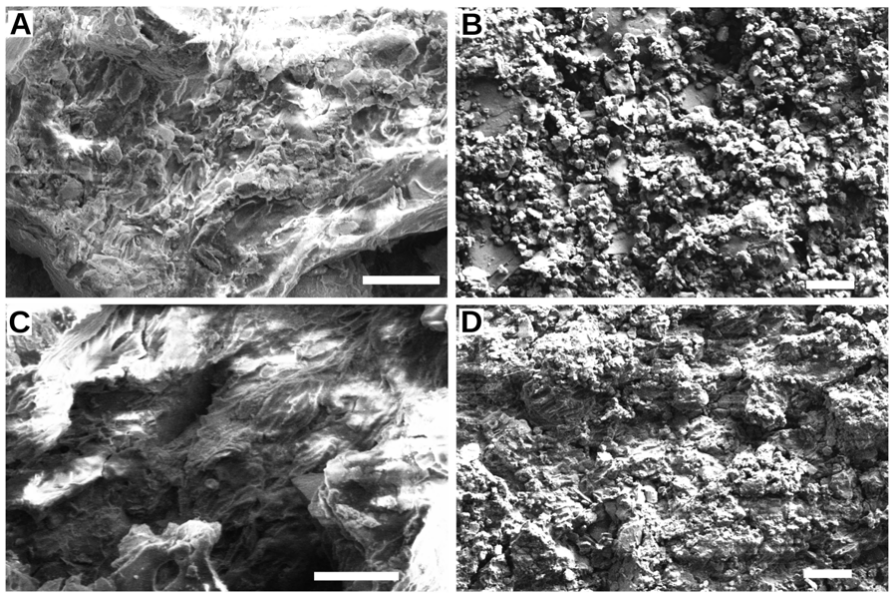
SEM images of *Gansus yumenensis* feathers and matrices. (A) MGSF317 and (B) MGSF318 matrices, and the light areas of feathers (C) MGSF317 and (D) MGSF318. Scale bars represent 4 µm.

Elongate mouldic structures 1.20 µm long and 0.26 µm wide (an average of 10 measurements, standard deviations 0.17 and 0.02 respectively) were observed in the extant Marabou stork feathers arranged regularly within fractured surfaces (Fig. 1C). No such structures were observed in the feathers of the extant White-naped Crane (Fig. 1D). Similar structures 1.66 µm long and 0.49 µm wide (an average of 10 measurements, standard deviations 0.16 and 0.07 respectively) were observed in dense patches on the dark areas of both fossil feathers (Fig. 1E–H), no such structures were seen in the light parts of the feathers or the matrix (Fig. 2). EDS analysis showed that the extant feathers are predominantly composed of carbon, oxygen and sulphur, though a significant amount of calcium (14.3 wt%) occurs in the Marabou stork feather but not in the White-naped Crane feather (Table 1). Analysis of the fossil feathers of MGSF317 and MGSF318 showed a much higher proportion of carbon in the dark areas (42.3 wt% and 19.0 wt% respectively) compared to the lighter areas (25.0 wt% and 5.4 wt% respectively) and the matrix (1.9 wt% in and 4.9 wt% respectively), which was mostly composed of silicon and oxygen as would be expected for sediment dominated by silicate minerals (Table 1). The calcium content of the dark area of the feather in MGSF317 is over twice that measured in lighter areas (10.5 wt% and 4.6 wt% respectively) however the opposite pattern was observed in the MGSF318 (Table 1).

**Table 1:** EDS determined elemental composition of extant and fossil samples show in weight percent.

	Weight percent (Wt. %)														
	C	O	F	Na	Mg	Al	Si	P	S	Cl	K	Ca	Ti	Fe	Cu
Marabou stork (*Leptoptilus crumeniferus*)	41.01	29.59	−	−	−	−	00.45	−	12.08	−	−	14.25	−	−	02.62
White-naped Crane (*Grus vipio*)	58.83	27.17	−	−	−	−	00.56	−	13.76	−	−	−	−	−	01.68
MGSF317 feather – dark part	42.74	31.83	00.08	05.44	02.42	00.36	00.76	00.10	00.82	03.42	00.01	10.48	00.03	00.84	00.67
MGSF318 feather – dark part	18.99	42.94	−	03.82	00.80	05.47	10.93	03.01	00.70	00.05	00.65	10.08	00.16	02.20	00.21
MGSF317 feather – light part	24.93	33.22	02.07	02.05	02.25	06.67	14.68	00.16	−	00.84	01.21	04.56	−	06.89	00.47
MGSF318 feather – light part	05.35	46.51	−	02.18	00.93	04.10	08.04	07.72	00.24	−	00.60	22.18	00.12	01.79	00.23
MGSF317 matrix	01.88	44.37	01.78	02.40	00.78	09.03	20.02	−	05.72	−	01.26	07.91	−	04.79	00.07
MGSF318 matrix	04.91	46.02	−	02.50	01.40	06.31	13.34	04.96	00.91	00.02	01.26	12.78	00.43	05.02	00.13

### FTIR

**Figure 3:**
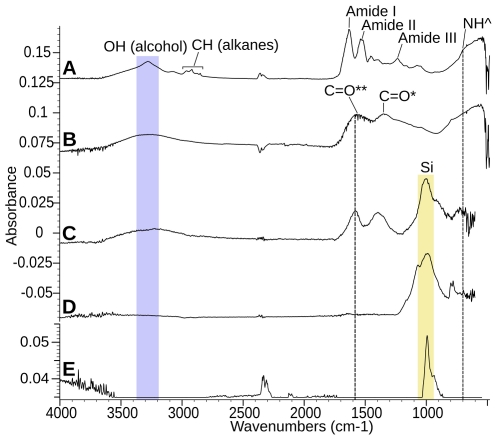
FTIR point analyses of *Sepia officinalis* eumelanin, *Gansus yumenensis* and extant feathers. (A) Extant Marabou stork feather, (B) *Sepia officinalis* eumelanin, (C) the dark area of an isolated *Gansus yumenensis* feather (MGSF317) and its associated matrix (D). *C = O represents a group from a carboxylic acid, **C = O represents a group from either a carboxylic acid or a ketone, NĤ represents a group from a secondary amine; inorganic peaks are shown with a yellow band. (E) Shows the resultant spectra when that of the natural *S. officinalis* melanin is subtracted from that taken from the dark area of the feather. Vertical dashed lines at 1582 cm^-1^ represent the C = O group of the ketone or asymmetric stretch of the carboxylic acid, and at 700 cm^-1^ represents the N-H wagging group of the secondary amine, the blue band represents the OH hydroxyl group.

**Figure 4:**
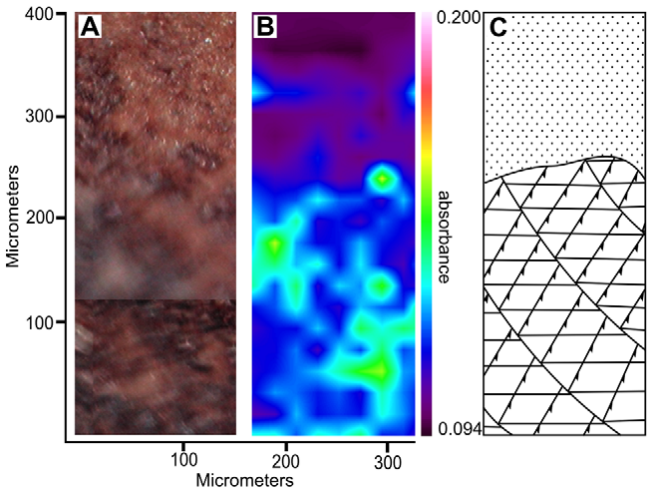
Infra-red absorption map of *Gansus yumenensis* barbules with corresponding visual image and diagram. (A) Optical microscope image of a section of barbules of a *Gansus yumenensis* feather (MGSF317) in reflected light, approximate position on the feather denoted by the yellow dot in figure 1B. (B) A map of infra-red absorption of the carboxylic acid C = O symmetric stretch at 1415 cm^-1^ of the whole optical image taken in ATR mode. (C) A diagrammatic interpretation of (A) shows the separation of feather barbules (bottom) and sedimentary matrix (top) to guide the eye.

Figure 3 presents infrared spectra of an extant feather (A), a eumelanin standard (B), a fossil feather (C) and its sedimentary matrix (D). The infrared spectra obtained from the fossil feathers were clearly different from those taken within the surrounding matrix. Because the spectra of both fossil specimens were so similar, only those of MGSF317 are shown in figure 3. The matrix showed only the presence of an inorganic silica band (Fig. 3D), whereas the fossil feathers also showed carboxylic acid, ketone, hydroxyl and potential secondary amine peaks (Fig. 3C, Table 2). The asymmetric C = O stretch and ketone C = O stretch are convolved in a single broad peak. We are also aware of the likelihood that additional C = C aromatic ring vibration signals may be convolved with both this peak and that of the C = O symmetric stretch [24]. These peaks all occur in the *Sepia officinalis* melanin spectra and the responsible functional groups are clearly seen in the chemical structure of eumelanin (Scheme 1b [25]). In addition, when the *Sepia officinalis* melanin spectrum (Fig. 3B) is subtracted from that of the fossil feathers (Fig. 3C) the only remaining peak is the inorganic silica band, i.e. all other peaks except atmospheric CO_2_ are accounted for by those of *Sepia officinalis* eumelanin (Fig 3E). A map of the absorbance of the carboxylic acid C = O symmetric stretch at 1622 cm^−1^ in MGSF317 (Fig. 4) shows good correlation of infra-red absorbance with visually dark areas of the feather. Melanin peaks were not resolvable in the spectrum of the extant Marabou Stork feather (Fig. 3A), this we attribute to the intense amide peaks of β-keratin overwhelming the signal from the melanin. The fossil feathers showed only extremely weak evidence of these amide peaks, seen as a subtle shoulder on the left of the carboxylic acid/ketone C = O peak, indicating that little IR reactive β-keratin has been preserved in comparison to the pigment. Details of peak assignments are given in Table 2.

**Table 2:** Infrared peak assignments for *Sepia officinalis* melanin, extant Marabou stork and White-naped Crane feathers and fossil *Gansus yumenensis* feathers MGSF317 and MGSF318.

Functional group	Bond	Wavenumber range (cm-1)	Intensity	Mode of vibration	MGSF318	MGSF317	Marabou stork feather (black)	White-naped Crane feather	Sepia melanin standard
Silicon compounds (Si-O)	Si-O	1100–1000	Strong	Stretching	—	—	X	X	—
	Si-O	990–945	Strong	Stretching	—	—	X	X	—
Ketone (C-(C = O)-C-OH)	C = O	1640–1540	Strong	Stretching	X	X	?	?	X
Carboxylic acid (COOH)	C = O	1600–1560	Strong	Asymmetric Stretching	X	X	?	?	X
	C = O	1420–1400	Strong	Symmetric Stretching	X	X	?	?	X
Hydroxyl (R-OH)	OH	3300–3280	Strong	Stretching	X	X	X	X	X
	OH	1450–1330	Medium	Deformation	X	X	X	X	X
	C-O	1100–1000	Strong	Stretching	X	X	X	X	X
Secondary amine (CH-NH-CH2)	N-H	3500–3300	Weak	Stretching	?	?	?	?	?
	N-H	1650–1550	Variable-weak	Deformation	?	?	?	?	?
	C-N	1191–1171	Weak	Stretching	?	?	?	?	?
	N-H	750–700	Strong	Wagging	?	?	?	?	?
Alkenes (C = C)	CH	3080–3000	Medium	Stretching	—	—	X	X	?
	CC	1690–1640	Medium	Stretching	—	—	X	X	?
Alkanes (R-CH3)	CH	2972–2952	Strong	Asymmetric stretching	—	—	X	X	—
	CH	2882–2862	Strong	Symmetric stretching	—	—	X	X	—
	CH	1475–1435	Medium	Asymmetric deformation	—	?	X	X	—
	CH	1380–1375	Medium	Asymmetric deformation	?	?	X	X	—
Alkanes (R′-CH2-R″)	CH	2936–2916	Strong	Asymmetric stretching	—	—	X	X	—
	CH	2863–2843	Strong	Symmetric stretching	—	—	X	X	—
	CH	1485–1445	Medium	Deformation	—	?	X	X	—
Amides (-CO-NH-C)	NH	3320–3270	Medium	Stretching	—	—	X	X	—
	C = O	1680–1630	Strong	Stretching	—	—	X	X	—
	CNH	1570–1515	Strong	Combination	—	—	X	X	—
	CNH	1305–1200	Medium-weak	Combination	—	?	X	X	—

### EPR

**Figure 5:**
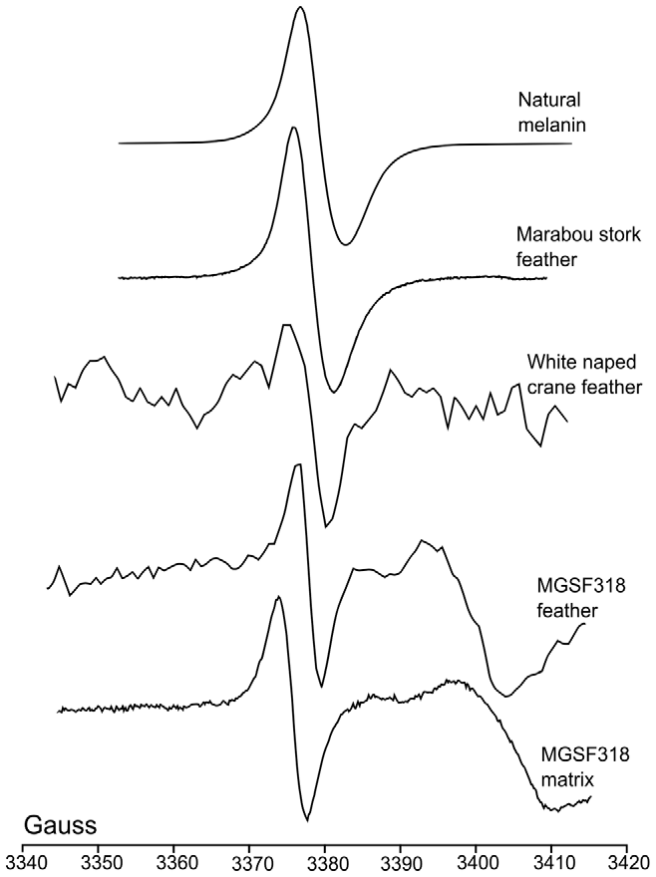
EPR spectra of *Sepia officinalis* eumelanin, *Gansus yumenensis* and extant feathers. Samples taken at room temperature. The Gauss scale (abscissa) is approximate due to the slight variations that occur in the frequency of the incident radiation; amplitudes are arbitrary.

The spectrum of the *Sepia officinalis* melanin showed a clear signal (Fig. 5) with a g value of 2.00421. This value fits well with the σ-semiquinone eumelanin signal identified in previous studies (g value between 2.0044 and 2.0030 [26]). A similar signal was present in the extant Marabou Stork feather (g value of 2.00418) and one was also detectable in the extant White-naped Crane feather (g value of 2.00357) but only in trace amounts, as seen in previous studies [27]. Due to limited sample volumes only the MGSF318 fossil could be analysed with EPR. The spectra from both MGSF318 matrix and feather samples showed an organic free radical signal as well as characteristic manganese hyperfine peaks (not shown). The calculated g value of the fossil feather sharp signal (2.00355) fell within the demonstrated σ-semiquinone g value range characteristic of eumelanin, whereas that of the matrix signal (2.00485) did not.

### Py-GCMS

**Figure 6:**
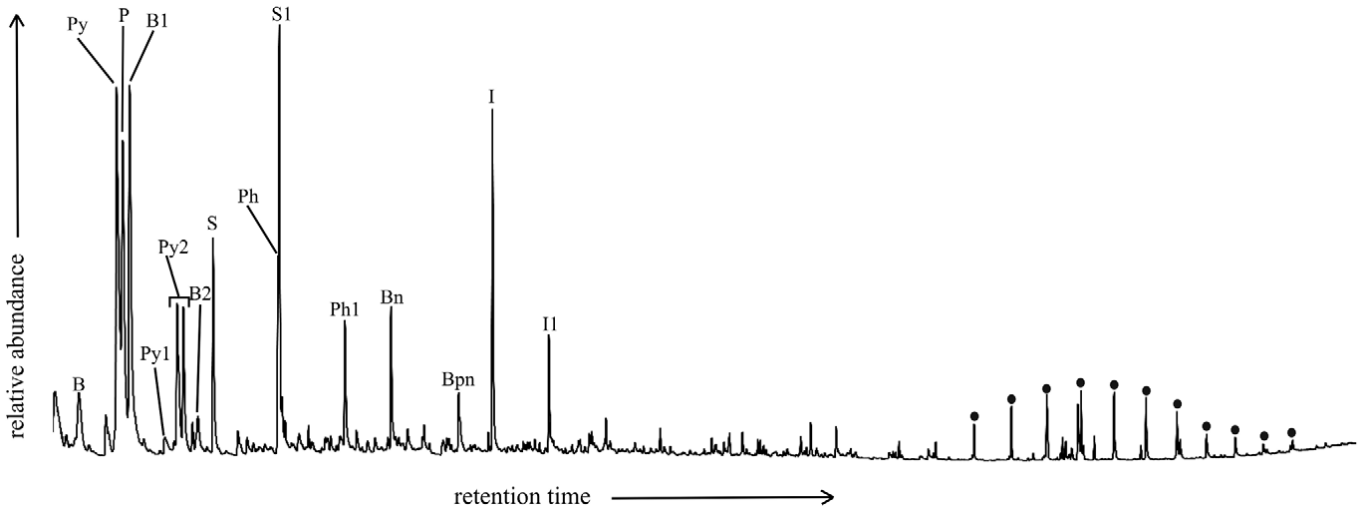
Partial ion chromatograph from the Pyrolysis GCMS of *Sepia officinalis* eumelanin. B: benzene, B1: methyl benzene, B2: ethyl benzene, Bn: benzene nitrile, Bpn: benzene propane nitrile, Py: pyrrole, Py1: pyrrole, 3-methyl, Py2: pyrrole, 2-methyl, P: pyridine, Ph: phenol, Ph1: phenol, 3-methyl, S: styrene, S1: methyl styrene, I: idole, I1: methyl indole.

**Figure 7:**
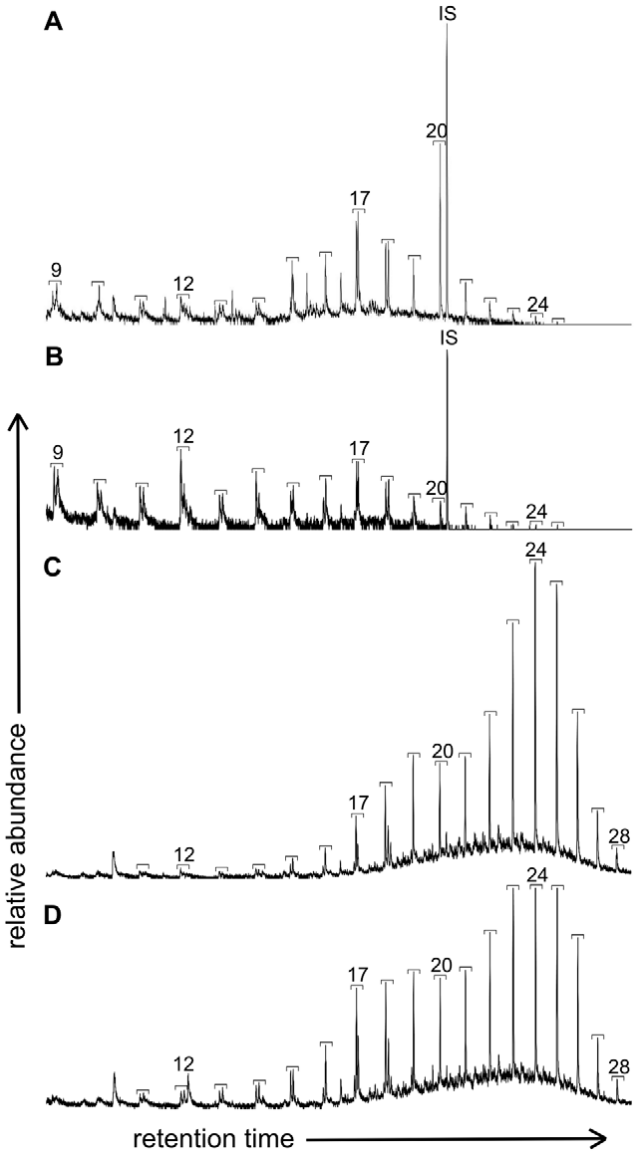
Partial Py-GCMS *m/z* 57 and 55 chromatograms of *Gansus yumenensis* feathers. (A) MGSF318 and (B) MGSF317 feathers, and their corresponding matrices (C and D respectively), showing the distribution of *n-*alkane/*n-*alkene moieties. Brackets indicate an *n-*alkane/*n-*alkene doublet and the numbers indicate the carbon chain length.

Analysis of the natural melanin sample produced a chromatogram containing mostly benzene, benzenenitrile, pyrrole and its methyl derivatives, phenol, phenol-4-methyl, styrene, indole and 3-methylindole (Fig. 6), comparable to previous studies [28], [29]. The total ion chromatograms of both fossil feather and matrix samples were dominated by a strong aliphatic signal and a series of benzene derivatives (not shown). Furthermore, comparison of partial *m/z* 57 and 55 mass chromatograms showed similar distribution patterns of *n-*alkanes/*n-*alkenes for both fossil feathers (a range from C_9_ to C_25_ with maxima at C_12_, C_17_ and C_20_, (Fig. 7A–B)), which differed from that of their corresponding sedimentary matrices (Fig. 7C–D). The abundances of *n-*alkanes/*n-*alkenes were higher in the matrices than the fossil feathers by a factor of 4 for MGSF317 and a factor of 2 for MGSF318. The MGSF318 matrix sample showed a bimodal distribution pattern dominated by peaks at C_24_ and C_19_; carbon chain lengths range from C_11_ to C_28_ (Fig. 7C). The matrix sample from MGSF317 also shows dominant peaks at C_24_ and C_19_, though the bimodal pattern is less pronounced; here carbon chain lengths again range from C_11_ to C_28_ (Fig. 7D). Both the matrix and feather samples of MGSF317 and MGSF318 contained benzene, toluene and styrene though the distribution patterns again varied between the feathers and their respective matrices (not shown).

## Discussion

SEM analysis of the *Gansus yumenensis* feathers demonstrates the presence of elongate structures that have been identified as eumelanosomes (Fig. 1E–H) after comparison with analysis of extant feathers (Fig. 1C) and previous studies [8]–[12]. These structures are confined to the visually darker areas of the fossil feather and were absent from both lighter areas and the matrix. The geochemical results from EPR, FTIR and Py-GCMS all show that the organic material in the feathers is different from that of their respective matrices, indicating that its source is the feather itself rather than contamination from the matrix. This can be readily seen in the infrared map of the melanic carboxylic acid group within the barbs of MGSF317 (Fig. 4), where the absorption pattern faithfully replicates the visually dark areas of the fossil feather. The infrared spectra of the fossil feathers are remarkably similar not only to each other, but also to the spectra of the natural eumelanin standard, as well as being starkly different from that of the matrix. The key functional groups (hydroxyl, ketone and carboxylic acid) are readily identified in both the spectra of the fossil feathers and natural eumelanin standard, and are clearly seen in the chemical structure of eumelanin (Scheme 1b [25]). There may be other molecules formed during decomposition that might also produce some of these functional groups. However, the fact that the functional groups characteristic of eumelanin are present in relative abundances comparable to those of this organic pigment makes it very likely that the fossil feather contains traces of eumelanin or eumelanin breakdown products. The Py-GCMS chromatograms show that the distribution patterns of aliphatics within the fossil feathers are very similar, whereas they are both different from their respective matrices. Previous experiments have shown that the origin of the aliphatic signal in fossil plants and insects is derived from the in-situ polymerization of labile lipid molecules [30], [31]. The aliphatic signal seen in the fossil feathers could be a consequence of a similar process though more experimental work is needed to confirm this. The signal in the matrix samples is most likely to come from other organic material present within the sediment, such as from plant material [31], [32]. No eumelanin pyrolysis products were observed in the chromatograms of the fossil feathers, nor indeed in the chromatograms of the extant feathers. In the case of the fossil feathers either the levels are below the detection limits of the instrument, or they are simply not detectable by this method in a specimen of this age. In the extant feathers the chromatograms showed mostly lipid and protein signals which likely overwhelmed any from the breakdown of eumelanin. The EPR data shows the presence of organic free radicals signals in both the MGSF318 matrix and the feather however these have measurably different g-values. The fossil feather has a g-value in accordance with that of the σ-semiquinone signal seen in the natural melanin standard whereas that of the sedimentary matrix does not, consistent with the presence of eumelanin within the fossil and not in the matrix. However we are cautious due to the low separation of these values and believe that on its own this does not constitute enough evidence to unequivocally identify eumelanin derived material within the fossil.

A key issue in this type of investigation is the problem of contamination, either ancient, modern or both. No bacterial hopanoid biomarkers were detected by the Py-GCMS analysis making contamination from modern bacteria unlikely, however the level of organic matter in the samples was too low for contributions from ancient bacterial biofilms to be detected. FTIR analysis revealed no bands characteristic of modern bacteria including CH bending from fatty acids or P-O-C and P-O-P stretching from phospholipids, ribose and phosphate chain pyrophosphate [33]. The dominant organic signals from bacteria are generally protein bands (amides I, II and III, 1655, 1546 and 1240–1235 cm^−1^ respectively) [33], [34]; whilst no distinct amide peaks are observed in the fossil feathers we cannot rule out the possibility that they occur in trace amounts due to the observation of a slight shoulder at 1660–1674 cm^−1^. However, amide bands may also be caused by the presence of keratin breakdown products derived from the feathers as recently demonstrated for fossil skin [32]. There are some types of bacteria that produce melanin [35], though we are currently unaware of any that are commonly associated with feathers; there are also some forms of soil fungi that produce melanin [36]. Though we see no evidence of either in the morphological or geochemical analyses we cannot definitively discount the possibility of their presence. Unfortunately due to the small sample sizes the number of destructive geochemical techniques we could perform were limited, this restricted the amount of information that could be gleaned from the sample. More investigation into the decomposition of fungi, bacteria and melanin would be useful and is the subject of future work. Human handling might be another potential source of contamination; however in this study every care was taken to avoid contamination by handling the samples with gloves, storing them in foil packets within a sealed plastic bag and also by taking the samples for EPR and Py-GCMS analysis under a fume hood. There is no chemical evidence of human contamination, no intact protein signals were detected by the FTIR and no cholesterol signals were visible in the Py-GCMS chromatograms. However yet again the most convincing argument against contamination, particularly human contamination, is not only that eumelanic functional groups faithfully replicate the darker areas of the feather, but also that the aliphatic signal is distinctly different in the matrix and the feathers. Our results are also in accordance with recent work demonstrating the use of trace metals, especially organically bound copper, within fossilized soft tissue as a biomarker for eumelanin presence within *Gansus yumenensis* [37]. The issue of contamination at the time of deposition by melanosomes leaching from decomposing skin has previously been raised [38], but the fact that the feathers are both isolated from any other soft tissue makes this highly unlikely. We note that there are possible transfer mechanisms even for an isolated feather, for example the feather could have been dislodged from a rotting carcass just before burial. However it is much more likely, given the evidence presented above, that the eumlenanin was endogenous.

Of the methods used in this study, only FTIR was both successful and non-destructive. Both EPR and Py-GCMS required the complete destruction of the samples for analysis, thus precluding the specimens from further future analysis. FTIR was able to identify eumelanic functional groups within the feathers and was also able to map their spatial distribution, clearly demonstrating their occurrence solely within the feather and not the matrix. Further organic geochemical analyses and studies into actualistic taphonomy must be done to help understand the preservation of organic material within fossil bacteria, this will assist in determining the extent of bacterial activity at the time of deposition.

### Conclusion

Current studies of purported melanosomes in fossil soft tissue solely rely upon morphological data to support their conclusions. Morphological evidence alone is insufficient to identify such structures with confidence, as has been found in similar studies of supposed Archean bacteria; “we caution against identifying microstructures as biological in origin without a full morphological and geochemical assessment” [39]. Here, in addition to morphological analysis, three geochemical analytical techniques were applied to *G. yumenensis* feathers to examine the preservation of any endogenous pigmentation. Structures were identified within the fossil feathers concordant with eumelanosomes comparable to structures seen in extant feathers as well as to previous studies of fossilized soft tissue. Infrared analysis strengthens this conclusion by demonstrating the presence of characteristic eumelanic functional groups within the fossil feather but not in the matrix, and these groups are shown to be spatially resolved with the feather material. EPR analyses proved unable to confidently distinguish characteristic melanin free radical signals within the fossil material despite showing the presence of different organic signals between feather and matrix. Pyrolysis GCMS clearly showed a difference between the organic material present within the fossil feathers as compared to their sedimentary matrices. There was also a clear similarity between the spectra of the two fossil feathers, implying that they may have a characteristic aliphatic signature. The combination of these techniques strongly suggests that there is both morphological and geochemical evidence for the preservation of original endogenous organic material and eumelanic pigment residue within the 105–115 million year old fossil feathers of *Gansus yumenensis*.
